# Proteomic features of skeletal muscle adaptation to resistance exercise training as a function of age

**DOI:** 10.1007/s11357-022-00658-5

**Published:** 2022-09-26

**Authors:** Colleen S. Deane, Bethan E. Phillips, Craig R. G. Willis, Daniel J. Wilkinson, Ken Smith, Nahoko Higashitani, John P. Williams, Nathaniel J. Szewczyk, Philip J. Atherton, Atsushi Higashitani, Timothy Etheridge

**Affiliations:** 1 Department of Sport and Health Sciences, College of Life and Environmental Sciences, University of Exeter, St. Luke’s Campus, Exeter, EX1 2LU UK; 2 Living Systems Institute, University of Exeter, Stocker Road, Exeter, UK; 3 Human Development & Health, Faculty of Medicine, University of Southampton, Southampton General Hospital, Southampton, UK; 4 School of Medicine, MRC-Versus Arthritis Centre for Musculoskeletal Ageing Research and National Institute for Health Research Nottingham Biomedical Research Centre, University of Nottingham, Derby, UK; 5 Department of Biomedical Sciences, Heritage College of Osteopathic Medicine, Ohio University, Athens, OH USA; 6 Ohio Musculoskeletal and Neurological Institute, Ohio University, Athens, OH USA; 7 School of Chemistry and Biosciences, Faculty of Life Sciences, University of Bradford, Bradford, UK; 8 Graduate School of Life Sciences, Tohoku University, 2-1-1 Katahira, Aoba-ku, Sendai, Miyagi 980-8577 Japan; 9 University Hospitals Derby & Burton NHS Foundation Trust, Royal Derby Hospital, Derby, UK

**Keywords:** Ageing, Network analysis, Hub protein, Proteomics, Phosphoproteome

## Abstract

Resistance exercise training (RET) can counteract negative features of muscle ageing but older age associates with reduced adaptive capacity to RET. Altered muscle protein networks likely contribute to ageing RET adaptation; therefore, associated proteome-wide responses warrant exploration. We employed quantitative sarcoplasmic proteomics to compare age-related proteome and phosphoproteome responses to RET. Thigh muscle biopsies were collected from eight young (25 ± 1.1 years) and eight older (67.5 ± 2.6 years) adults before and after 20 weeks supervised RET. Muscle sarcoplasmic fractions were pooled for each condition and analysed using Isobaric Tags for Relative and Absolute Quantification (iTRAQ) labelling, tandem mass spectrometry and network-based hub protein identification. Older adults displayed impaired RET-induced adaptations in whole-body lean mass, body fat percentage and thigh lean mass (*P* > 0.05). iTRAQ identified 73 differentially expressed proteins with age and/or RET. Despite possible proteomic stochasticity, RET improved ageing profiles for mitochondrial function and glucose metabolism (top hub; PYK (pyruvate kinase)) but failed to correct altered ageing expression of cytoskeletal proteins (top hub; YWHAZ (14–3-3 protein zeta/delta)). These ageing RET proteomic profiles were generally unchanged or oppositely regulated post-RET in younger muscle. Similarly, RET corrected expression of 10 phosphoproteins altered in ageing, but these responses were again different vs. younger adults. Older muscle is characterised by RET-induced metabolic protein profiles that, whilst not present in younger muscle, improve untrained age-related proteomic deficits. Combined with impaired cytoskeletal adhesion responses, these results provide a proteomic framework for understanding and optimising ageing muscle RET adaptation.

**Supplementary Information:**

The online version contains supplementary material available at 10.1007/s11357-022-00658-5.

## Introduction

Chronological ageing is characterised by progressive loss of skeletal muscle mass and function (‘sarcopenia’) [1], which associates with increased risk of frailty-related falls [2], morbidity [3] and, ultimately, mortality [4]. The most effective non-pharmacological intervention to maintain/increase muscle mass and strength across the life course is resistance exercise training (RET) [5]. However, older age associates with impaired capacity for muscle adaptation in response to RET [6]. Exploring the mechanisms underpinning age-related reductions in adaptive capacity stands to promote optimised RET strategies and new sarcopenia therapeutic discovery [7], but these remain incompletely defined.

In recent years, large-scale ‘omic’ analyses have been increasingly applied to characterise molecular regulators of RET adaptation across age, focussing predominantly on gene transcriptomic changes. Such studies identify impaired transcriptional responses in mitochondrial [8, 9], extracellular matrix and immune system genes [10] as defining features of age-related anabolic resistance to RET. However, alternative splicing patterns of individual genes, plus several other post-transcriptional events, can give rise to multiple, functionally diverse protein products. The protein translational machinery is also highly dynamic, and the half-life of genes versus proteins differs significantly. Gene expression changes do not, therefore, inform clearly on the biochemical mediators responsible for cell functional changes and fail to reliably associate with corresponding protein abundance [11, 12]. Indeed, RET responsive genes involved in anabolic signalling do not always induce comparable protein changes in young and older people [13]. As the functional units of cells, altered protein expression must ultimately underpin (ab)normal muscle adaptation [14]; thus, proteome-level responses to RET across age should be established. Nonetheless, proteomic adaptations to RET are underexplored [15], with just two studies examining age-related RET proteome profiles [13, 16] and one study comparing the myofibrillar and non-myofibrillar proteomes in younger-trained, younger-untrained and older-untrained volunteers [17]. Robinson et al. [13] reported that 12 weeks whole-body RET improved the mitochondrial proteome in older people and increased protein translation/ribosomal protein content, indicative of improved translational capacity. In ageing rodents, 12 weeks of RET increased glycolytic protein expression in gastrocnemius muscle [16]. As such, further research is needed to improve protein-level resolution of RET-induced ageing muscle regulation to provide the basis for intervention optimisation and expedite drug discovery routes.

Most human muscle proteomic profiling has employed two-dimensional gel electrophoresis (2DGE) which, despite high resolution [18], typically associates with low reproducibility [19] and underrepresentation of proteins possessing extreme isoelectric points, certain post-translation modifications, high molecular mass or low abundance [20]. Technological advances have permitted a shift away from gel-based-proteomics towards ‘shotgun’ based proteomics including quantitative peptide identification methods such as Isobaric Tags for Relative and Absolute Quantification (iTRAQ) [21]. iTRAQ labels all peptides via free amines at their N-terminus and lysine side chains. By exploiting up to 8 different isobaric tags, iTRAQ offers improved peptide identification sensitivity through summation of mass peaks across pooled samples. Subsequent fragmentation of tag-specific reporters, each possessing differing isotope distribution, permits the ratio of signal intensities to be interpreted as relative peptide content between differently labelled samples [22–24]. Although its use in the skeletal muscle field is scarce, iTRAQ proteomic analysis of interval exercise trained, young, healthy muscle found increased content of mitochondrial proteins involved in oxidative phosphorylation and fatty acid metabolism [25]. Given the potential power of quantitative proteomics, here we applied iTRAQ proteomics/phosphoproteomics and network-driven analysis to older muscle vs. younger counterparts before and after chronic RET, to establish protein network profiles that characterise ageing muscle responses to RET.

## Materials and methods

### Volunteer characteristics and ethics

Skeletal muscle samples and corresponding physiological data from eight young (age: 25.0 ± 1.1 years; gender: 4 males, 4 females; body mass index: 22.8 ± 0.8 kg/m^2^) and eight older (age: 67.5 ± 2.6 years; gender: 6 males, 2 females; body mass index: 27.7 ± 0.8 kg/m^2^) adults were randomly selected from a larger RET intervention study cohort previously published by our laboratory [8, 26]. All volunteers were screened via medical questionnaire and physical examination, which included resting electrocardiogram, blood pressure assessment and blood biochemistry. Volunteers were excluded if they had: overt muscle wasting (> 2 standard deviations below age norms [27]), metabolic, respiratory or cardiovascular disorders or any other signs/symptoms of ill-health. Volunteers were recreationally active but did not routinely participate in moderate/high intensity aerobic exercise and had not participated in RET in the previous 2-year period. All study-associated risks and procedures were thoroughly explained to volunteers and written informed consent was obtained prior to participation. This study was reviewed and approved by the University of Nottingham Faculty of Medicine and Health Sciences Research Ethics Committee (D/2/2006) and was conducted in accordance with the Declaration of Helsinki.

### Study design

Volunteers took part in a fully supervised, progressive, whole-body RET programme, as previously described [8, 26]. Volunteers trained for 20 weeks in total, performing 3 training session per week, with each session lasting ~ 60 min, in line with previous recommendations designed to achieve muscle hypertrophy and enhance muscle strength [28]. During the initial 4 weeks of RET, intensity was increased from 40 to 60% 1-repitition maximum (1-RM), to allow adoption and adherence to correct technique. Thereafter, training intensity was set at 70% 1-RM for the remaining 16 weeks, with multiple sets of 12 repetitions and 2 min rest between sets. Every session consisted of the same number of repetitions for 8 different exercises (totalling 16 sets of 12 repetitions): latissimus pull-down, seated chess press, seated lever row, leg curl, leg extension, leg press, abdominal curl and back extension. To ensure the intensity of training remained constant, 1-RM assessments were conducted every 4 weeks. Volunteers would have been excluded from the study for non-compliance, defined as non-attendance for > 5 consecutive sessions, less that 75% total attendance, or failure to complete the set-exercise regime in 15% sessions; however, compliance was 100%.

A skeletal muscle biopsy (~ 150 mg) was obtained from the *m. vastus lateralis* pre- and post-RET in the fasted, non-exercised state, using the conchotome biopsy technique [29] under local anaesthetic (2% lidocaine). Skeletal muscle tissue was rapidly dissected free of visible fat and connective tissue, rinsed in ice-cold phosphate buffered saline, blotted on gauze, immediately snap frozen in liquid nitrogen and stored at − 80 °C for subsequent proteomic analysis. The post-RET muscle biopsy was obtained ~ 72 h after the final RET session to specifically determine the RET effect on the proteome.

### Protein extraction

Proteomics was performed on the sarcoplasmic muscle fraction, isolated from ~ 20 mg of frozen muscle biopsy tissue. Muscle was rapidly homogenised with scissors in ice-cold extraction buffer (10 µL mg^−1^) containing a complete protease inhibitor cocktail tablet (Roche, West Sussex, UK), 50 mM Tris–HCl (pH 7.4), 0.1% Triton X-100, 1 mM EDTA, 1 mM EGTA, 50 mM NaF and 0.5 mM activated sodium orthovanadate (Sigma Aldrich, Poole, UK). Homogenates were centrifuged at 13,000 rpm for 5 min at 4 °C. The resultant sarcoplasmic supernatant of age and time-point matched samples were combined to yield a total of 4 samples suitable for iTRAQ 4-plex, that is: 8 young pre-RET samples were combined to form 1 young pre-RET sample, 8 older pre-RET samples were combined to form 1 older pre-RET sample, 8 young post-RET samples were combined to form 1 young post-RET sample, and 8 older post-RET samples were combined to form 1 older post-RET sample. Protein concentration of the sarcoplasmic supernatants was determined using the Bradford assay. For each sample, 20 µg total protein was precipitated in six volumes of cold acetone at − 20 °C for 2 h, and then centrifuged at 10,000 g for 1 min with the supernatant discarded and the precipitated pellet used for subsequent digestion and iTRAQ labelling.

### Protein digestion and iTRAQ labelling

Protein digestion and iTRAQ labelling was performed in line with manufacturer instructions (AB SCIEX, Foster City, CA, USA). In brief, to reduce proteins, 20 µl of dissolution buffer (containing 0.5 M triethylammonium bicarbonate, pH 8.5) and 1 µl of denaturant (containing 2% sodium dodecyl sulphate) was added to each sample and vortex mixed, followed by 2 µl of reducing agent (containing 50 mM tris-(2-carboxyethyl)phosphine), vortex mixing and incubation at 60 °C for 1 h. Cysteine blocking was performed by adding 1 µl cysteine blocking reagent (containing 200 mM methyl methanethiosulfonate in isopropanol), vortex mixing and incubation at room temperature for 10 min. Proteins were then digested by incubating samples with trypsin overnight at 37 °C followed by centrifugation to pellet the protein digest. Peptides were subsequently labelled by adding iTRAQ reagent (amine-modifying labelling reagent) to each sample and vortex mixing. pH was checked to ensure samples were pH 7.5–8.5 for optimal labelling efficiency.

To understand average proteomic responses across conditions, all 8 volunteer samples for young pre-RET, older pre-RET, young post-RET and older post-RET were pooled to provide a single labelled sample per condition and labelled with iTRAQ reagents 114, 115, 116 and 117, respectively. As such, any change in peptide abundance observed, represents a composite of changes across all 8 volunteers. This approach was chosen to avoid the technical variance in peptide labelling and MS/MS peptide identification that would arise with an experimental design that assigned a single iTRAQ label to each individual muscle sample (which, with a maximum 8-plex available, would require comparisons of proteomic experiments across 4 separate technical runs for our dataset). Despite this pooled approach not allowing for analyses of the inter-individual responses to ageing and RET, it does provide good power for identifying generalised peptide abundance changes across all volunteers. Samples were incubated at room temperature for 2 h before being combined into one sample tube. The combined sample mixture was then cleaned free of substances that can interfere with mass spectrometry analysis (e.g. triethylammonium bicarbonate, sodium dodecyl sulphate) using cation-exchange chromatography (Vivapure S Mini H, Sartorius), eluted with 100 mM or 1 M KCl and 10 mM potassium phosphate in 20% (v/v) acetonitrile at pH 3.0 (Cation-Exchange system Buffers, ABsciex), and then each fraction was desalted (Sep-Pak C18 Plus Light Cartridge, Waters, Milford, MA, USA).

### LC-MD/MS analysis

Phosphopeptides were enriched using a Titansphere Phos-TiO kit (GL Sciences, Japan). After an evaporation to remove the acetonitrile, aliquots were loaded onto a C18 tip column and stored at − 80 °C until used as described [30]. Mass spectrometry was performed using TripleTOF™ 5600 System (ABsciex) with an Eksigent ekspert™ nanoLC column, 3 μm, ChromXP C18-CL, with a 111 min (for total peptides) and a 65 min (for phosphopeptides) gradient going from 2 to 90% acetonitrile, 0.1% formic acid and a 300 nL/min flow. During acquisition, each survey scan accumulated precursors in the range of 400–1250 m/z for 250 ms (for total peptides) and the range of 100–1600 m/z for 200 ms (for phosphopeptides).

### Protein identification and quantification

Protein identification and iTRAQ-based relative quantification were both undertaken using the ProteinPilot™ Software (version 4.0; AB Sciex Inc., USA). Protein identification was performed by searching MS/MS spectra against the NCBI human database using the Paragon™ Algorithm [31], with the thorough search effort setting applied and search parameters set to indicate tryptic digestion and allow for cysteine alkylation by methyl methanethiosulfonate, biological modifications and amino acid substitutions. During identification the detected protein threshold was set at 1.3 (95% detection confidence), with bias and background correction both employed and false discovery rate (FDR) analysis also integrated into the search phase by means of a target-decoy strategy. Protein GI accessions were consequently mapped to associated gene symbols using the Universal Protein Resource (UniProt) knowledge database [32]. Relative quantification of proteins was determined based on the ratio of peak areas from MS/MS spectra, with protein expression fold-changes and associated *P* values calculated in each of the following cases: old baseline (i.e. pre-RET) vs. young baseline (i.e. pre-RET); young baseline vs. young post-RET; and older baseline vs. older post-RET. Protein hits occurring in the reverse library, suspected contaminants (haemoglobin and keratin proteins) and proteins identified by less than 2 peptides were thereafter excluded from further analyses. Remaining proteins were then screened for differential regulation foremost by fold-change (with cut-offs of > 1.2 and < 0.8 used to define differential upregulation and downregulation, respectively) and secondarily by statistical significance (*P* < 0.05) or a trend towards statistical significance (*P* ≤ 0.1).

### Functional annotation and classification

The functional characteristics of differentially expressed proteins were elucidated by testing relevant protein lists for both Gene Ontology (GO) term enrichment and biological pathway enrichment. Analysis was undertaken using the Enrichr web server [33], with the Biological Process (BP), Cellular Component (CC) and Molecular Function (MF) categories each considered for GO term enrichment, and both the Kyoto Encyclopaedia of Genes and Genomes (KEGG) and Reactome databases considered in the case of pathway enrichment. The Benjamini–Hochberg procedure was used to control for FDR and GO pathway terms with an FDR < 1% were consequently defined as significantly enriched.

### Network-driven analyses

Network-based analysis of relevant differentially expressed protein lists was undertaken using the NetworkAnalyst online resource [34]. In each case, a first-order skeletal muscle tissue-specific proteome network was initially constructed to model interactions between differentially expressed proteins and all other proteome components that directly interact with them, using parameters as previously described [9]. Each first-order network was thereafter transformed into a minimum interaction network, whereby only differentially expressed proteins and those proteome components needed to maximally connect them were kept. The highly connected ‘hub’ proteins of each minimum interaction network were consequently deduced based on betweenness centrality (a measure of how often a given node lies on the shortest path between two other given nodes), such that the top 5 components of a given network when ranked by betweenness centrality were defined as hub proteins. Hub proteins with an equal betweenness centrality ranking were additionally prioritised by node degree (i.e. number of connections to other nodes). Relevant network visualisations were generated using Cytoscape (v3.7.1) [35].

### Phosphopeptide analysis

Phosphopeptides were normalised to total protein using ProteinPilot™. Non-phosphorylated peptides, mis-allocated peptides (according to ProteinPilot™) and very low confidence peptides (< 10) were removed. Data was analysed using Student’s *t*-tests and was accepted as significant if *P* ≤ 0.05. Data are presented as mean ± SEM.

### Statistical analysis

For the physiological measures, unpaired Student’s *t*-tests were used to determine basal differences. Two-way repeated measures mixed-effects analysis with Sidak’s multiple comparison analysis were used to determine age × time effects. Data analysis was conducted using GraphPad Prism version 9 (GraphPad Software, San Diego, CA). Data were accepted as significant if *P* < 0.05 and are presented as mean ± SEM.

## Results

### Physiological adaptations to RET

Demonstrating age-related adaptive deficits to RET, whole-body lean mass increased (*P* = 0.01), body fat percentage decreased (*P* = 0.0004), and the increase in single upper leg lean mass approached significance (*P* = 0.06) in young, but not older adults following RET, as assessed by dual-energy X-ray absorptiometry (Table 1). Confirming the effectiveness of the progressive training programme, RET increased lower-body strength in both young and older adults (*P* < 0.005). Compared to young adults, older adults displayed higher fasting glucose levels pre-RET (*P* = 0.03), indicative of impaired glucose handling, which was reversed by RET (*P* = 0.04). Fasting insulin and HOMA were unaffected (*P* > 0.05). Mean arterial pressure, total cholesterol and low-density lipoprotein levels were significantly higher in older adults pre- and post-RET (*P* < 0.05), reflecting an age-related increase in cardiovascular disease risk, and were unaffected by RET in either group (*P* > 0.05). There was no impact of age or RET on basal muscle fractional synthesis rate (*P* > 0.05, methods described in (25)). Individual data points are shown in Fig. S1.

**Table 1:** Physiological measurements pre and post 20 weeks of supervised resistance exercise training in young and older adults (mean ± SEM)

	Young		Older	
	Pre-RET	Post-RET	Pre-RET	Post-RET
*Body composition*				
Lean body mass (kg)	47.97 ± 3.87	49.06 ± 4.11*	52.82 ± 4.61	53.04 ± 4.29
Lean leg mass (kg)	8.83 ± 0.56	8.67 ± 0.61	8.91 ± 0.93	9.21 ± 0.80
Upper lean leg mass (kg)	4.55 ± 0.46	4.61 ± 0.54^~^	4.83 ± 0.46	5.09 ± 0.47
Relative skeletal muscle index^a^ (kg/m)	28 ± 1	28 ± 2	30 ± 2	30 ± 2
Body fat (%)	30 ± 5	26 ± 4*	33 ± 2	32 ± 2
*Function*				
Whole-body strength (N)	4,328 ± 388	6,147 ± 471*	4,198 ± 590	5,622 ± 725*
Lower-body strength (N)	2,538 ± 195	3,664 ± 219*	2,462 ± 375	3,197 ± 368*
*Metabolism*				
Fasting insulin (µUnits/ml)	4.79 ± 0.85	4.59 ± 0.27	4.83 ± 0.81	4.23 ± 0.73
Fasting glucose (mmol/L)	5.06 ± 0.18^	4.97 ± 0.20	6.00 ± 0.20^	5.43 ± 0.38*
HOMA	1.07 ± 0.18	1.02 ± 0.08	2.21 ± 0.58	1.06 ± 0.25
Basal FSR (% h^−1^)	0.046 ± 0.007	0.058 ± 0.009	0.044 ± 0.005	0.052 ± 0.009
*Cardiovascular*				
Systolic blood pressure (mmHg)	116 ± 4^	115 ± 4^	138 ± 7^	135 ± 5^
Diastolic blood pressure (mmHg)	67 ± 3	78 ± 3*	70 ± 3	79 ± 3*
Mean arterial pressure (mmHg)	98 ± 3^	99 ± 3^	117 ± 5^	115 ± 4^
Resting heart rate (bpm)	66 ± 3	61 ± 3	59 ± 4	57 ± 3
Total cholesterol (mmol/L)	3.56 ± 0.20^	3.70 ± 0.26^	5.39 ± 0.27^	4.89 ± 0.25^
High density lipoprotein (mmol/L)	1.21 ± 0.13	1.27 ± 0.11	1.33 ± 0.06	1.22 ± 0.12
Low density lipoprotein (mmol/L)	1.94 ± 0.17^	2.05 ± 0.18^	3.61 ± 0.30^	3.14 ± 0.15^
Triglycerides (mmol/L)	0.89 ± 0.10	0.81 ± 0.07^	1.00 ± 0.10	1.15 ± 0.04^

*A significant difference from baseline within age (*p* < 0.05); ^~^a close to significant difference from baseline within age (*p* = 0.06); ^a significant difference between groups at that time point (*p* < 0.05). ^a^Whole-body strength was determined by the sum of force produced by three lower-body exercises and three upper-body exercises, with newtons calculated as weight lifted × 9.807 based on a standard gravitational field [26]

### Protein expression changes with ageing and/or RET

**Fig. 1:**
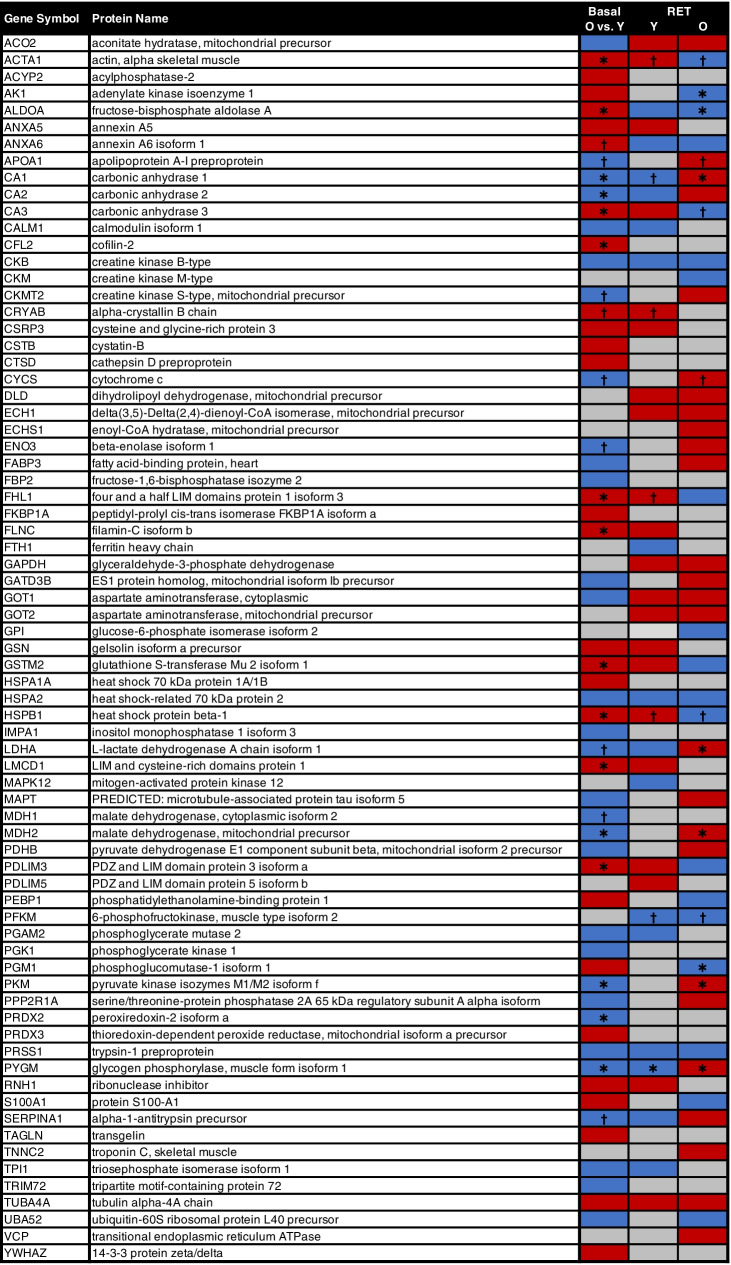
Protein expression changes with ageing and/or resistance exercise training. Shown are all proteins with an expression fold-change > 1.2 or < 0.8 in old (O) vs. young (Y) muscle at baseline (basal), and/or following resistance exercise training (RET) in young muscle and/or in old muscle (vs. respective baselines). In each given instance, red and blue shading denote upregulation and downregulation, respectively. *P < 0.05, ^**†**^*P* ≤ 0.1

**Fig. 2:**
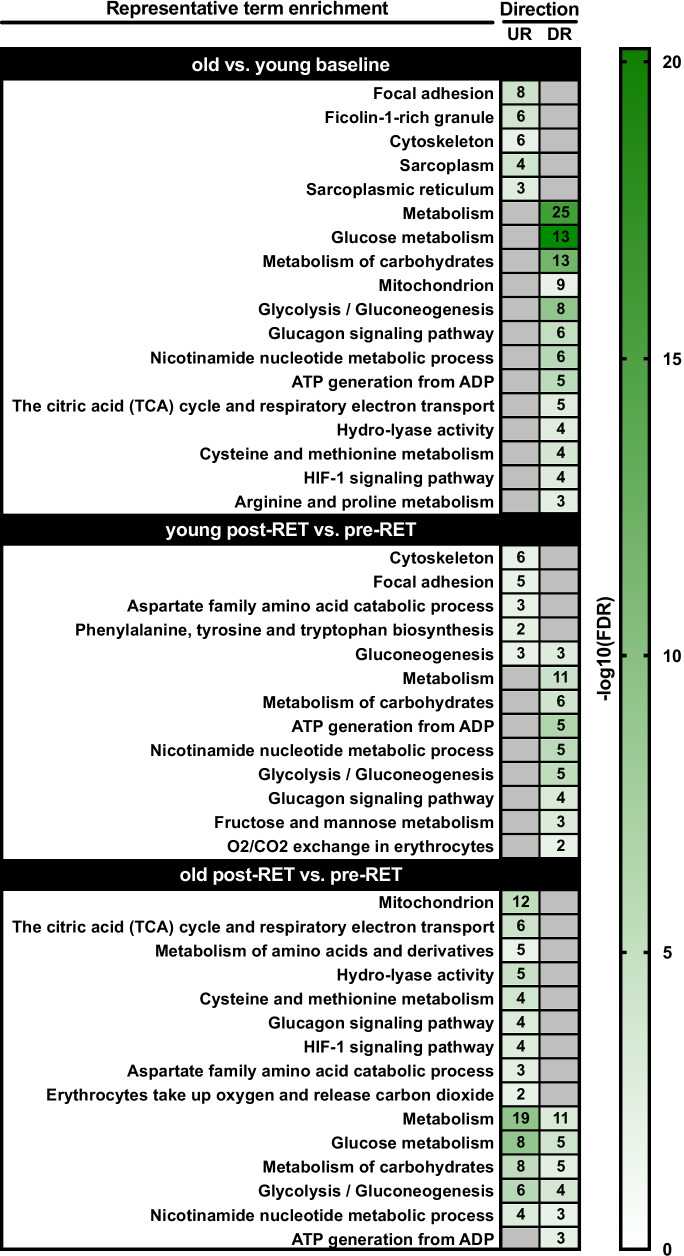
Functional characteristics of differentially expressed proteins. Given are representative enriched Gene Ontology and biological pathway (KEGG/Reactome) terms for differentially upregulated (UR) and downregulated (DR) protein lists in each of the following cases: (i) old vs. young baseline; (ii) post-resistance exercise training (RET) vs. baseline (i.e. pre-RET) in young muscle; and (iii) post-RET vs. baseline in old muscle. Strength of colour shading depicts magnitude of enrichment significance, given by the negative log10 of that term’s enrichment false discovery rate (FDR) *P*-value (with darker shading analogous with a stronger FDR *P*-value). Numbers of enriched proteins in each case are given in associated heatmap boxes

In total, 164 proteins were detected for downstream bioinformatics, similar to previous proteomic work [16, 24], with 73 proteins displaying differential expression as a function of age and/or in response to RET (Fig. 1). Of the 164 identified, only 9.7% had < 10% sequence coverage across a minimum of 2 peptide sequences (Tab s1). To identify age-related differences in the basal skeletal muscle proteome, we compared differentially expressed proteins from young and older baseline (i.e. pre-RET) muscle. We found 29 proteins demonstrating increased expression in older muscle, which were predominantly enriched for muscle structural terms such as focal adhesion, cytoskeleton and sarcoplasmic reticulum (Fig. 2). A similar number of proteins (31) demonstrated decreased expression in older muscle (Fig. 1), which were largely enriched for energy metabolism related terms, and in particular glucose-related metabolism and NAD^+^/mitochondrial oxidative phosphorylation processes (Fig. 2).

To understand how RET alters the muscle proteome, we next directly compared differentially expressed proteins between pre- to post-RET muscle within age groups. In young adults, RET induced an upregulation of 21 proteins largely related to cytoskeleton, focal adhesion and amino acid metabolism, and a downregulation of 16 proteins strongly related to glucose metabolism (Figs. 1 and 2). Conversely, in older adults RET induced an upregulation of 26 proteins predominantly related to glucose metabolism and mitochondrial function (including citric acid cycle, respiratory electron transport). However, in this age-group, RET also downregulated 19 proteins strongly related to glucose metabolism (Figs. 1 and 2). This, perhaps, reflects a degree of age-associated proteomic stochasticity in ageing RET responses, with greater degrees of both up- and downregulated proteins identified under multiple ‘metabolic’ protein functional terms versus consistent expression change directionality in young RET proteomic profiles.

### Comparing age-related RET proteomic profiles

**Fig. 3:**
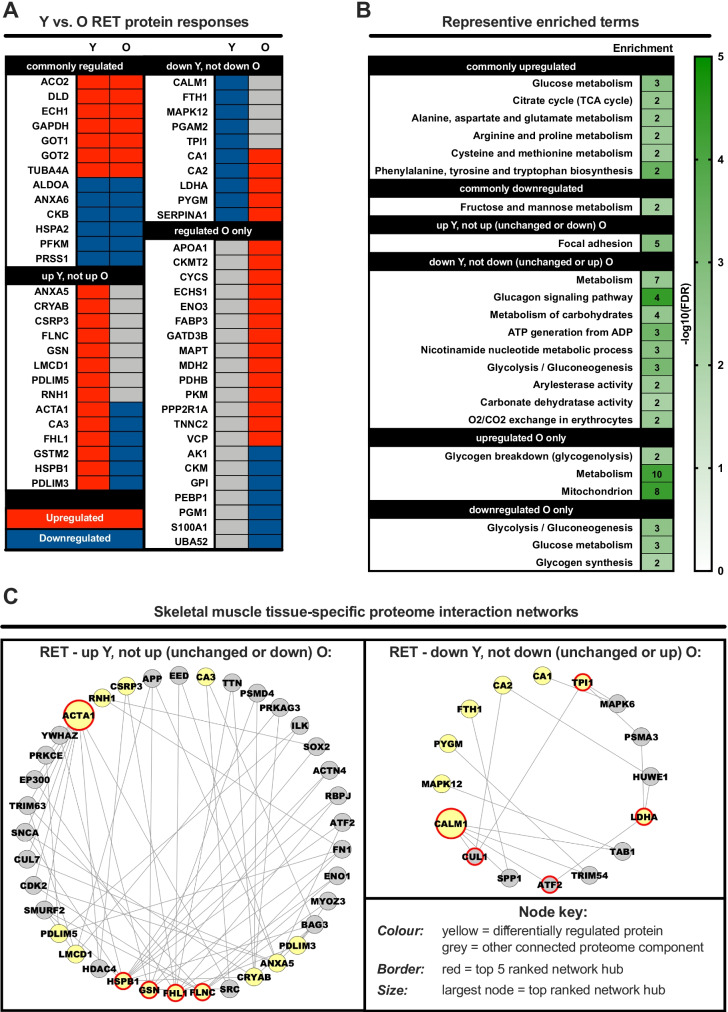
Direct comparison of protein expression changes in young vs. old muscle following resistance exercise training. Panel **A**: heatmap depicting the similarities/differences in protein expression changes induced by resistance exercise training (RET) in old (O) vs. young (Y) muscle. Red and blue shading denote upregulation and downregulation, respectively. Panel **B**: representative enriched Gene Ontology and biological pathway (KEGG/Reactome) terms for the various different permutations depicted in panel **A**. Strength of colour shading depicts magnitude of enrichment significance, given by the negative log10 of that term’s enrichment false discovery rate (FDR) *P*-value (with darker shading analogous with a stronger FDR *P*-value). Numbers of enriched proteins in each case are given in associated heatmap boxes. Panel **C**: skeletal muscle-specific minimum interaction networks for proteins regulated by RET in young muscle that are not regulated in the same manner by RET in old muscle. In each case, yellow nodes represent differentially regulated proteins whilst grey nodes represent other strongly interacting components of the proteome. Red borders depict hub proteins in each instance, with the top ranked hub given by a larger node size

Next, we directly compared young vs. older RET proteomic profiles to identify common and/or unique age-related RET responses (Fig. 3A, Tab s2). Independent of age, RET mostly increased the expression of proteins involved in amino acid-related metabolism, whilst downregulating proteins involved in fructose and mannose metabolism, perhaps suggesting these processes fundamentally contribute to RET-induced remodelling across the lifespan (Fig. 3B). Moreover, functional terms for glycolysis/glucose metabolism were downregulated in both young and older muscle post-RET, albeit separate proteins comprised these terms for each age group (Fig. 3B). Proteins upregulated uniquely in young muscle post-RET were related to focal adhesions, whereas proteins increased only in older muscle post-RET were enriched for generic metabolism functional terms, glycogenolysis and mitochondrial function (Fig. 3B).

We then generated minimum protein interaction networks and used hub protein analysis to determine key proteins putatively driving the RET response. We identified ACTA1, FHL1, FLNC, GSN and HSPB1 as hub proteins within the minimum protein interaction network constructed from proteins upregulated by RET in young muscle only, with ACTA1 being the top ranked hub protein (Fig. 3C). For the network constructed using proteins downregulated by RET in young muscle, CALM1, LDHA, TPI, CUL1 and ATF2 were identified hub proteins, with CALM1 being the top ranked hub protein (Fig. 3C, Tab s3).

### RET-induced effects on age-related protein profiles

**Fig. 4:**
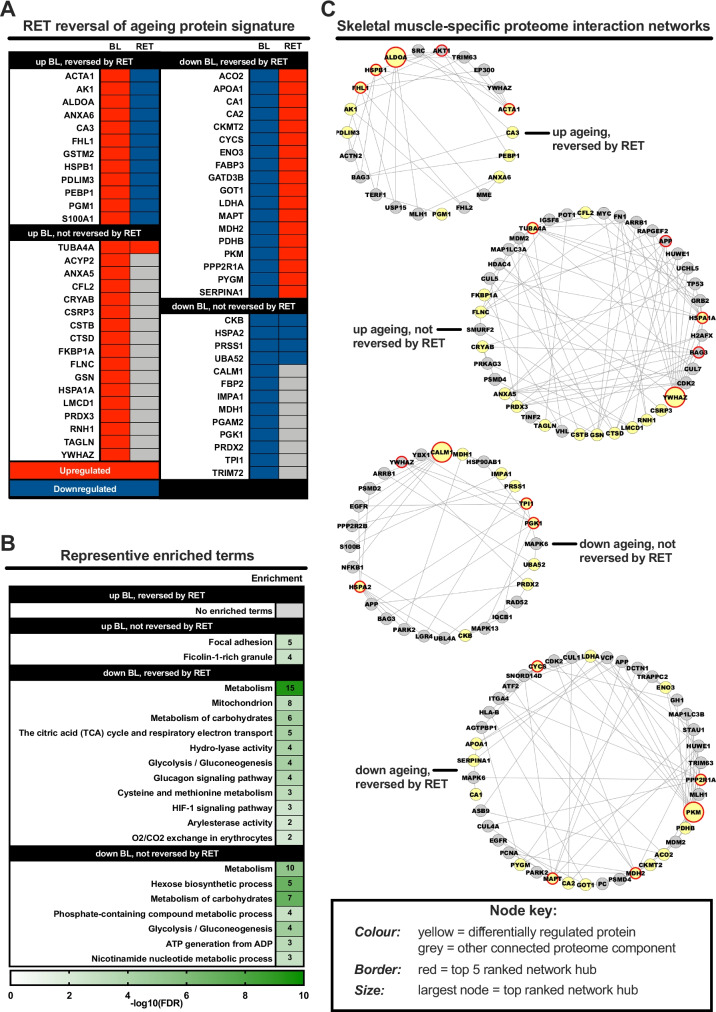
Capacity for resistance exercise training to reverse age-related protein dysregulation. Panel **A**: heatmap depicting the degree by which resistance exercise training (RET) reverses protein dysregulation with ageing per se. That is, how proteins dysregulated in old vs. young muscle at baseline (BL) respond to RET in old muscle. Red and blue shading denote upregulation and downregulation, respectively. Panel **B**: representative enriched Gene Ontology and biological pathway (KEGG/Reactome) terms for the various different permutations depicted in panel **A**. Strength of colour shading depicts magnitude of enrichment significance, given by the negative log10 of that term’s enrichment false discovery rate (FDR) *P*-value (with darker shading analogous with a stronger FDR *P*-value). Numbers of enriched proteins in each case are given in associated heatmap boxes. Panel **C**: skeletal muscle-specific minimum interaction networks for proteins dysregulated by ageing that do/do not favourably respond to RET in old muscle. In each case, yellow nodes represent differentially regulated proteins whilst grey nodes represent other strongly interacting components of the proteome. Red borders depict hub proteins in each instance, with the top ranked hub given by a larger node size

Finally, we to sought to understand whether RET can reverse the altered baseline (i.e. pre-RET) ageing muscle proteome. To achieve this, we compared dysregulated proteins in older baseline muscle (i.e. proteins up- or downregulated vs. younger baseline muscle) to the corresponding post-RET signature for each protein(s) (Fig. 4A). Proteins upregulated at baseline and subsequently downregulated by RET were not enriched for any functional terms, but did include metabolic-related (e.g. AK1, CA3, ALDOA) and LIM domain (FHL1, PDLIM3) proteins (Fig. 4A B). RET reversed the basal downregulation of several proteins predominantly clustered to several metabolism functional terms, namely the mitochondrion and glucose-related metabolic processes (Fig. 4B). However, not all metabolism-related proteins suppressed with age were reversed by RET, including those involved in hexose biosynthetic processes, NAD^+^ metabolism and other metabolism-related terms. RET also failed to lower the expression of proteins upregulated at baseline in ageing muscle, namely proteins related to focal adhesion and ficolin-1-rich granule (i.e. cell adhesion processes). All enriched terms are provided in Tab s2.

At the network level, ALDOA, HSPB1, FHL1, ACTA1 and AKT1 were identified as hub proteins within the network constructed from proteins with increased basal expression in older muscle and reversed by RET, with ALDOA being the top ranked hub. Hub proteins of the network constructed from proteins upregulated with ageing at baseline but not reversed by RET include YWHAZ (top ranked), HSPA1A, TUBA4A, APP and BAG3. Hub proteins of the network constructed from proteins downregulated with ageing and reversed by RET are PKM, PPP2R1A, MAPT, MDH2 and CYS, with PKM as the top ranked hub. Hub proteins of the network constructed from proteins downregulated with ageing but not reversed by RET are CALM1, TPI1, PGK1, HSPA2 and YWHAZ, with CALM1 being top ranking hub protein*.* All associated network statistics are provided in Tab s3.

### Phosphoproteomic changes with ageing and RET

**Fig. 5:**
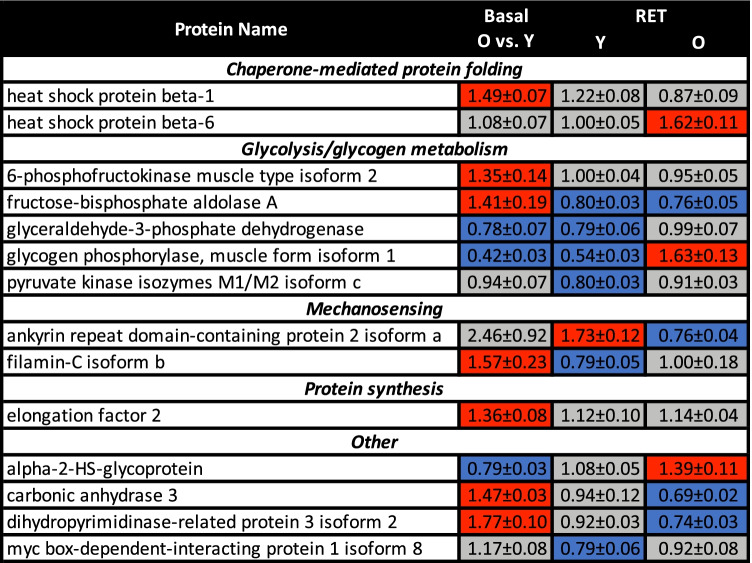
Phosphoproteomic expression changes with ageing and/or resistance exercise training. Shown are all phosphoproteins which display significant upregulation (red shading) and/or downregulation (blue shading) in old (O) vs. young (Y) muscle at baseline (basal), and/or following resistance exercise training (RET) in young muscle and/or in old muscle (vs. respective baselines) (*P* ≤ 0.05). Data are mean ± SEM

Our phosphoproteomic approach identified 40 phosphopeptides from iTRAQ labelled samples, 14 of which displayed altered expression with age and/or RET. The majority of these were related to chaperone-mediated protein folding, glycolysis, mechanosensing and protein synthesis (Fig. 5, Tab s4). Consistent with our proteomic data, older basal muscle was characterised by increased phosphorylation of heat shock protein beta-1 (HSPB1), filamin-C isoform b (FLNC) and carbonic anhydrase 3 (CA3). Additionally, glycogen phosphorylase muscle isoform 1 (PYGM) displayed downregulation by RET in young muscle and upregulation following RET in older muscle, whereas CA3 phosphorylation was lowered in older muscle following RET. Of the 11 RET-responsive phospho-protein changes, only one target displayed the same downregulated response in both young and older people (fructose-bisphosphate aldolase A); all 10 other phospho-proteins displayed divergent responses in older muscle.

## Discussion

RET remains the cornerstone of treatment for offsetting sarcopenic progression, yet impaired adaptive responses with advancing age, through incompletely defined mechanisms, assigns scope for RET optimisation in older people. Here, we utilised quantitative proteomics and network-driven analysis to establish (phospho)protein networks and hub proteins that characterise older muscle responses to 20 weeks supervised RET. Our findings reveal that age-related glucose and mitochondrial protein profiles are at least partially reversed by RET, but that these RET profiles, including observed phosphoproteomic changes, are generally different to profiles of younger people. The inability of focal adhesion proteins to appropriately respond to RET in older muscle might also contribute towards age-related adaptive deficits.

Lowered content of mitochondrial proteins typified untrained older muscle, mirroring previous reports of age-related reductions in mitochondrial gene/protein expression [13] and oxidative capacity [36, 37]. Ageing further associates with a fast-to-slow twitch muscle fibre transition [38], a phenomenon supported by reproducible increases in slow-fibre enriched proteins, such as carbonic anhydrase 3, observed herein and previously [17, 18, 38]. Thus, compromised oxidative capacity, despite a relative shift towards a slow-twitch phenotype might represent a common functional feature of ageing muscle. After RET, the content of these mitochondrial proteins was increased, in line with previous reports of training-induced gains in mitochondrial proteins in older populations using unbiased [13] and targeted Western blot approaches [39]. Within this mitochondrial network, cytochrome c (CYCS) and malate dehydrogenase 2 (MDH2) emerged amongst the top ageing-RET hub proteins. CYCS transfers electrons between complexes III and IV and is released to the cytosol to signal caspase-mediated cell death [40]. MDH2 effects reversible oxidation of malate to oxaloacetate, coupled with oxidation of NADH to NAD^+^ [40]. These ageing, RET-responsive hubs therefore reflect important mitochondrial oxidative and cell signalling roles that, along with their interaction networks, provide interesting targets for promoting ageing muscle maintenance. Additionally, although direct increases in expression of components of NAD^+^ metabolism did not occur after RET, other proteins within the NAD^+^ pathway did show increased abundance after RET in ageing muscle. A general pattern of enhanced mitochondrial capacity therefore characterises ageing RET adaptations, which does not feature strongly in younger muscle, supporting previous observations [41, 42].

The untrained ageing muscle proteome and phosphoproteome also associated with upregulation of focal adhesion-related and cytoskeletal protein networks. This finding supports increasing evidence that elevated focal adhesion/cytoskeletal expression [43] and phosphorylation [44] is a fundamental compensatory mechanism of ageing muscle attempting to counteract losses in the myofibrillar network [45]. Paradoxically, we [46] and others (e.g. [47]) report that increased focal adhesion expression signatures also typify the normal RET response of younger healthy muscle. This consistent molecular phenomenon across inconsistent phenotypes (i.e. older muscle decline and younger muscle hypertrophy) underscores the highly complex and dynamic nature of adhesion-mediated muscle maintenance exhibited across species [48–51]. Determining the precise nature of beneficial vs. harmful muscular consequences of upregulated focal adhesions should prove fruitful for understanding and countering muscle loss across the life course, for which our list of eight focal adhesion proteins increased with ageing could provide a relevant foundation.

Moreover, RET was unable to lower age-related focal adhesion expression. A model might, therefore, exist whereby younger muscle experiences RET-induced increases in content and/or phosphorylation of multiple focal adhesion components, facilitating muscle adaptive remodelling via mechanotransduction [52]. Conversely, increased adhesion protein expression with advancing age, whilst initially compensatory, may diminish RET-related responsiveness, and subsequent failure within the complex three-dimensional functional adhesome [48] cannot effectively mechanically mediate muscle adaptive signalling. This, in turn, could be a central factor governing reduced ageing muscle RET adaptations. Whilst key regulatory focal adhesion proteins are undefined, our network analysis implicates the 14–3-3 protein YWHAZ as the top ranked hub within proteins increased with ageing but unaltered post-RET. YWHAZ is a highly conserved transmembrane protein that recognises serine and threonine phosphorylation sites of numerous binding partners to affect wide-ranging signalling pathways, the precise muscular role of which in ageing and RET adaptations could be explored. Additionally, the molecular chaperone HSPA1A also emerges from the top 5 ranked hub proteins for this network, and HSP1 displays elevated phosphorylation with age. Given the role of HSP1A in nascent protein folding and stabilisation of proteins to prevent toxic aggregation, its deregulation with age and unresponsiveness to RET might also be an interesting target of future research.

Impaired glucose handling is a well-documented feature of ageing muscle [53], and our untrained older volunteers exhibited elevated fasting blood glucose levels. This was also reflected at the proteome level, with reduced glucose metabolism-related proteins and phosphopeptide profiles presenting as prominent features of older muscle. RET increased much of this ageing proteomic and phosphoproteomic glucose mishandling profile, which appears functionally relevant with ageing fasting blood glucose levels correspondingly improved post-RET. The ageing RET protein network also highlighted pyruvate kinase isozymes M1/M2 isoform f (PKM) [53] as the top hub protein upregulated by RET. In addition to its glycolytic function, the PKM interaction network communicates diverse non-glycolytic functions [53], and might represent an important hub coordinating RET-induced metabolic responses in older adults. However, whilst these signatures appear beneficial for glucose handling in older muscle, glucose-related protein and phosphoprotein signatures of younger muscle generally responded to RET in an opposite, downregulatory manner. RET adaptations in the glucose-enzyme axis [54] might, therefore, rely on augmented glycogen substrate availability [55] rather than increased glycolytic enzyme activity/content. Additionally, whilst RET specifically increased abundance of ageing-supressed glucose metabolism proteins (e.g. PKM, PPP2R1A, ENO3, GOT1, MDH2, PYGM), several other proteins clustering to the same glucose functional terms displayed post-RET reductions (e.g. TPI1, MDH1, PGAM2, PGK1, CAALM1, UBA52, FBP2). Considering the highly consistent directionality of glucose-related protein expression changes in younger muscle, irregular directional changes in older people might indicate that the stochastic theory of natural ageing [56, 57] could also extend to ageing responses to RET. Individualised exercise prescription strategies might, therefore, be highly relevant for optimising training adaptations in older cohorts.

Coverage of the proteome using iTRAQ was limited, albeit in line with previous studies using this technique [16, 24], and iTRAQ is known to result in a ~ 20% reduction in the number of differentially expressed peptides identified vs. unlabelled methods [58, 59]. Nonetheless, this was sufficient for downstream bioinformatic analysis and identification of distinct differences in proteomic signatures between young and older people pre- and post-RET that support previous label-free studies (e.g. [18, 38]). Small sample numbers also increase the risk of false associations but, despite only analysing 32 muscle samples, these derive from a highly controlled, fully supervised RET intervention trial linked to robust physiological phenotyping. This data should, therefore, contribute to the wider systems biological goal of understanding the complicated array of molecular drivers underpinning muscle adaptation in health and disease. Our approach of pooling samples for each condition precludes person-specific associations between proteomic changes and functional outcomes. Alternative labelling approaches or unlabelled experiments are, therefore, required to determine proteomic signatures that might explain intraindividual differences in muscle adaptations to RET. Nonetheless, quantitative proteomics identified (phospho)proteomic signatures and putative hub candidates driving older muscle responses to RET. Untrained, age-related reductions in metabolic protein profiles respond positively to RET but (a) appear to exhibit some post-RET stochasticity and (b) are insufficient to fully prevent functional deficits in older muscle. Indeed, these upregulated metabolic signatures are generally different to younger muscle, which display opposite downregulation post-RET. Ageing also associates with increased focal adhesion/cytoskeletal protein content that is not responsive to RET and thus impaired adaptability of mechanotransduction pathways might be an important driver of impaired adaptability of older muscle to RET.

## Supplementary Information

Below is the link to the electronic supplementary material.

Supplementary file1 (XLSX 30 KB)

Supplementary file2 (XLSX 25 KB)

Supplementary file3 (XLSX 16 KB)

Supplementary file4 (XLSX 53 KB)

Supplementary file5 (DOCX 165 KB)
