# Supplementary material for: Proteomic features of skeletal muscle adaptation to resistance exercise training as a function of age
Source: GeroScience. 2022 Sep 26;45(3):1271–87. doi: 10.1007/s11357-022-00658-5 (PMC10400508; doi:10.1007/s11357-022-00658-5)
Supplement: Supplementary file 5 — Supplementary file5 (DOCX 165 KB) [file 11357_2022_658_MOESM5_ESM.docx]

**Fig s1: Raw data points for physiological measurements pre and post 20 weeks of supervised resistance exercise training in young and older adults.** A) lean body mass, B) lean leg mass, C) upper lean leg mass, D) relative skeletal muscle index, E) body fat, F) whole-body strength, G) lower body strength, H) fasting insulin, I) fasting glucose, J) HOMA, K) basal FSR, L) systolic blood pressure, M) diastolic blood pressure, N) mean arterial blood pressure, O) resting heart rate, P) total cholesterol, Q) high density lipoprotein, R) low density lipoprotein and S) triglycerides. FSR, fractional synthesis rate. HOMA, homeostatic model assessment, RET, resistance exercise training. * denotes a significant difference from baseline within age (*p*<0.05); ^~^ denotes a close to significant difference from baseline within age (*p*=0.06); ^ denotes a significant difference between groups at that time point (*p*<0.05).
